# Health-related quality of life of the parents of children hospitalized due to acute rotavirus infection: a cross-sectional study in Latvia

**DOI:** 10.1186/s12887-018-1086-y

**Published:** 2018-03-15

**Authors:** Gunta Laizane, Anda Kivite, Inese Stars, Marita Cikovska, Ilze Grope, Dace Gardovska

**Affiliations:** 1 Department of Paediatrics, Riga Stradins University, Vienibas gatve 45, Riga, LV-1004 Latvia; 2 Department of Public Health and Epidemiology, Riga Stradins University, Kronvalda boulevard 9, Riga, LV-1010 Latvia

**Keywords:** Rotavirus gastroenteritis, Health-related quality of life, Latvia, Childhood, Acute, Impact, Family

## Abstract

**Background:**

Rotavirus is the leading cause of severe diarrhea in young children and infants worldwide, representing a heavy public health burden. Limited information is available regarding the impact of rotavirus gastroenteritis on the quality of life of affected children and their families.

The objectives of study were to estimate the impact of rotavirus infection on health-related quality of life (HRQL), to assess the social and emotional effects on the families of affected children.

**Methods:**

This study enrolled all (*n* = 527) RotaStrip®-positive (with further PCR detection) cases (0–18 years of age) hospitalized from April 2013 to December 2015 and their caregivers. A questionnaire comprising clinical (filled-in by the medical staff) and social (filled by the caregivers) sections was completed per child.

**Results:**

Main indicators of emotional burden reported by caregivers were compassion (reported as severe/very severe by 91.1% of parents), worry (85.2%), stress/anxiety (68.0%). Regarding social burden, 79.3% of caregivers reported the need to introduce changes into their daily routine due to rotavirus infection of their child. Regarding economic burden, 55.1% of parents needed to take days off work because of their child’s sickness, and 76.1% of parents reported additional expenditures in the family’s budget.

Objective measures of their child’s health status were not associated with HRQL of the family, as were the parent’s subjective evaluation of their child’s health and some sociodemographic factors. Parents were significantly more worried if their child was tearful (*p* = 0.006) or irritable (*p* < 0.001). Parents were more stressful/anxious if their child had a fever (*p* = 0.003), was tearful (*p* < 0.001), or was irritable (*p* < 0.001). Changes in parents’ daily routines were more often reported if the child had a fever (*p* = 0.02) or insufficient fluid intake (*p* = 0.04).

**Conclusion:**

Objective health status of the child did not influence the emotional, social or economic burden, whereas the parents’ subjective perception of the child’s health status and sociodemographic characteristics, were influential.

A better understanding of how acute episodes affect the child and family, will help to ease parental fears and advise parents on the characteristics of rotavirus infection and the optimal care of an infected child.

## Background

Rotavirus is known to be the leading cause of severe gastroenteritis among infants and young children worldwide [1]. Rotavirus gastroenteritis is frequently associated with severe disease symptoms (vomiting, diarrhea, dehydration, etc.) and increased hospitalization episodes compared to other types of acute gastroenteritis caused by infectious agents [2].

Rotavirus gastroenteritis represents a heavy public health burden [3]. From 2010 to 2015, an average of 3000 registered rotavirus cases per year are reported in the age group of 0–6 years, being responsible for an average of approximately 1000 hospitalizations per year in Latvia [4].

The epidemiology of rotavirus gastroenteritis is well documented [5], but these data are not the only indicators of disease burden. Limited information is available regarding the impact of rotavirus gastroenteritis on the quality of life of affected children and their families [5].

Health-related quality of life (HRQL) refers to the subjective and objective impact of dysfunction associated with an illness or injury, medical treatment, and health care policy [6] and integrates physical, emotional and social well-being and functioning as perceived by the individual [7]. In pediatric research, HRQL measure has received an increasing attention and is recognized as a substantial health outcome [8]. Pediatric HRQL research is necessary to examine broader psychosocial outcomes and provide an in-depth understanding of the effects of disease and treatment on children’s health status [9]. Nerveless, this measure is primarily used in children with various chronic diseases [8].

In the case of pediatric disease, assessment of HRQL of the family is becoming increasingly important because a child’s illness affects the whole family as a holistic system. Studies in this area provide information on family needs, responses to the child’s disease, coping strategies and changes in family functioning. Most studies are related to childhood chronic diseases, such as congenital heart disease [7], bleeding disorders [10], atopic dermatitis [11], attention deficit/hyperactivity disorder [12], chronic kidney disease [13], and juvenile idiopathic arthritis [14], etc., in association with the quality of family life because of the long-term progression of such diseases and their impact on quality of life.

Less information is available regarding associations between temporary health conditions, such as acute rotavirus gastroenteritis, and HRQL. However, as childhood rotavirus gastroenteritis is a public health problem, it should be evaluated beyond clinical trials with respect to the psychological, social and economic consequences of the disease.

Studies that evaluated the effect of acute childhood rotavirus gastroenteritis on the family have revealed negative effects on family function and parental psycho-emotional wellbeing [5, 15–17]. Parents indicated economic impact, such as lost work days lost due to the child’s disease [5] and additional direct costs [17], disruption of schedules and restrictions on daily activities [15–17], high distress and worries due to symptoms [5, 15–17], exhaustion and helplessness [16], need for additional childcare and the use of more nappies [5].

The aim of this study was to estimate the impact of rotavirus infection on HRQL and to assess the social and emotional impacts on the families of affected children. In addition, the factors associated with HRQL characteristics will be clarified.

This article reports the family impact of rotavirus gastroenteritis requiring hospitalization of a child based on individual interviews with parents or legal caregivers and objective data from patient files.

## Methods

### Study design

To investigate the quality of life of families where child is suffering from acute rotavirus infection, a quantitative cross-sectional study was carried out among caregivers of children who had been hospitalized in the Children’s Clinical University Hospital in Riga from April 2013 to December 2015.

### Inclusion and exclusion criteria

The study enrolled all hospital cases of rotavirus-positive children (0–18 years of age) and their caregivers (parents or legal family representatives). Caregivers had to be willing to participate and provide written consent. As exclusion criteria included the absence of caregivers or caregivers not providing signed consent.

### Data collection

Parents, of the laboratory confirmed rotavirus positive children, were invited to participate in individual interviews. The interviewer collected data regarding the clinical status of the child from patient files, and interviewed parents about emotional, social and economic factors pertaining to their child affecting their daily lives. All results and answers were collated in a questionnaire.

### Instruments used

A questionnaire was developed to estimate the impact of rotavirus infection on parents of affected children.

The questionnaire consisted of two general parts: clinical (filled-in by the medical staff) and social (filled by the caregivers) parts. The clinical part posed questions regarding the demographic data of the patient and family, and objective and subjective signs and symptoms to determine the clinical severity of the case. To categorize clinical severity, the Vesikari score [18] was used. The social part of the questionnaire was developed based on concepts and research methods used in previous similar studies [5, 15–17] and covered the following domains of the impact of pediatric rotavirus on the family: 1) parental emotional wellbeing and feelings (distress; helplessness; mental exhaustion; worry; anxiety for the child; fear of being infected; feelings of guilt); 2) social burden of disease (or the disease impact on parents’ daily activities (work schedule, training plans (syllabus), leisure time activities, domestic works (household)); 3) economic burden of the disease (working days lost due to child disease, additional financial expenditures); 4) parental opinion about the child’s physical symptoms (diarrhea, vomiting, fever, abdominal pain, dehydration, loss of appetite) and changes in behavior (apathy, sleeping disorders, irritability, anxiety); 5) parental opinion about rotavirus vaccine use (awareness of vaccine existence (yes/no); use of vaccine (yes/no; if answered “no”, the parents were asked about their motives for refusal).

Five-hundred twenty-seven hospitalized RotaStrip®-positive subjects further confirmed by PCR were enrolled in the study from April 2013 to December 2015. Totally 3301 hospitalized cases were registered from 2013 to 2015. As all enrolled patients were rotavirus-positive, the study did not have a rotavirus negative control group, but that can be considered in future research.

### Statistical analysis

Descriptive statistics such as means for continuous variables and proportions for categorical variables were calculated. To evaluate the statistical significance of the differences of proportions of severe/very severe cases between subgroups, a Chi-square test or Fisher’s exact test were used. Statistical significance was set at *p* = 0.05.

Data processing was performed using IBM SPSS Statistics (Statistical Package for the Social Science, Version 22.0).

## Results

### Demographic characteristics of study subjects and their parents

The characteristics of the subjects and their parents are summarized in Table 1 (uploaded as separate file). The children’s mean age was 26.1 months, and the sex ratio was balanced between male and female subjects. The majority of responding parents where in the 25–34 year-old age group. Collected data on education levels revealed that majority of mothers had a higher education; among fathers - persons with secondary/vocational education and a higher education were equally represented. Most respondents had a stable social status, and were living in urban areas. Low income citizens are defined by Cabinet of Ministers of Latvia by regulation No.299. It determines that citizens with total monthly income less than 128.06 EUR per family member, can obtain status of low income person, and may apply for social support. Others have stable social status [19].

**Table 1:** Demographic and clinical characteristics of the study subjects and their parents (*n* = 527^a^)

Parameter	Number	Percent
Age of the child (months)		
Mean (range)	26.1 (1–209)	
≤ 12	156	29.7
13–24	168	31.9
25–36	89	16.9
≥ 37	113	21.5
Gender of the child		
Female	258	49.0
Male	269	51.0
Age of the mother (years)		
≤ 24	55	10.5
25–34	335	63.8
35–44	127	24.2
≥ 45	8	1.5
Age of the father (years)		
≤ 24	27	5.3
25–34	281	55.1
35–44	164	32.2
≥ 45	38	7.5
Education of mother		
Primary	29	5.6
Secondary/vocational	189	36.2
Higher	304	58.2
Education of father		
Primary	36	7.2
Secondary/vocational	245	48.7
Higher	222	44.1
Place of residence		
Urban	449	87.2
Rural	66	12.8
Social status		
Low-income	28	5.4
Socially stable	491	94.6

^a^The sum of the stratified numbers can differ according to the parameters due to missing values

### Objective and subjective appraisal of child’s health status

Clinical symptoms were categorized as severe according to the Vesikari score [18] in 93% patients (*n* = 463) and moderate in 7% (*n* = 35); no mild cases were detected. The objective and subjective appraisals of the health status of the included children are summarized in Table 2 (uploaded as separate file). Three symptoms most often notified by parents as very severe were diarrhea (mentioned by 53.6% (*n* = 280) of parents), insufficient fluid intake (49.6%, *n* = 259) and loss of appetite (41.5%, *n* = 215).

**Table 2:** Objective and subjective appraisal of the child’s health status (*n* = 527^a^)

Parameter	Number	Percent
Maximal number of vomiting episodes per day		
Mean (range)	2.1 (0–3)	
Number of diarrhea episodes per 24 h		
Mean (range)	2.5 (1–3)	
Severity (assessed by Vesikari score)		
Mild	0	0
Moderate	35	7.0
Severe	463	93.0
Severity of symptoms (assessed by parent)		
Diarrhea		
Not at all	16	3.1
Mild	17	3.3
Moderate	77	14.8
Severe	132	25.3
Very severe	280	53.6
Vomiting		
Not at all	82	15.7
Mild	61	11.7
Moderate	87	16.7
Severe	111	21.3
Very severe	181	34.7
Fever		
Not at all	78	15.0
Mild	65	12.5
Moderate	110	21.2
Severe	109	21.0
Very severe	158	30.4
Abdominal pain		
Not at all	92	18.1
Mild	71	14.0
Moderate	135	26.6
Severe	108	21.3
Very severe	102	20.1
Insufficient fluid intake		
Not at all	40	7.7
Mild	34	6.5
Moderate	84	16.1
Severe	105	20.1
Very severe	259	49.6
Loss of appetite		
Not at all	45	8.7
Mild	44	8.5
Moderate	106	20.1
Severe	108	20.5
Very severe	215	41.5
Apathy		
Not at all	43	8.4
Mild	42	8.2
Moderate	106	20.6
Severe	139	27.0
Very severe	184	35.8
Inflamed bottom		
Not at all	203	39.2
Mild	81	15.6
Moderate	87	16.8
Severe	67	12.9
Very severe	80	15.4
Interrupted sleep mode		
Not at all	167	32.2
Mild	94	18.1
Moderate	121	23.3
Severe	82	15.8
Very severe	55	10.6
Tearfulness		
Not at all	78	15.0
Mild	75	14.4
Moderate	153	29.4
Severe	131	25.2
Very severe	83	16.0
Anxiety, irritability		
Not at all	124	23.9
Mild	95	18.3
Moderate	121	23.4
Severe	104	20.1
Very severe	74	14.3

^a^The sum of the stratified numbers can differ according to the parameters due to missing values

### Assessment of emotional, social and economic impact of the disease on the family quality of life

Emotional, social, and economic impact of the disease is summarized in Table 3 (uploaded as separate file). Speaking about emotional burden of rotavirus infection - a very high level of compassion was found, mentioned as very severe in 76.4% (*n* = 402) of questionnaires, followed by a very high level of worry in 59.6% (*n* = 311) of cases and stress/anxiety (37.8% (*n* = 199) of cases). Social burden was analyzed by changes in daily routines, and the analyzed data showed that 79.0% (*n* = 413) of families had changes in their daily routine. Economic impact was analyzed by describing parental work day loss directly related to episodes of their child’s illness. It revealed that only 33.1% (*n* = 173) of parents did not need to take any days off work. Additionally - 75.2% (*n* = 380) of respondents had extra expenditures due to the disease (symptomatic drugs, diapers, etc.).

**Table 3:** Assessment of emotional, social and economic impact of the disease on the family quality of life (*n* = 527^a^)

Parameter	Number	Percent
Emotional burden		
Stress, anxiety		
Not at all	15	2.9
Mild	46	8.7
Moderate	112	21.3
Severe	154	29.3
Very severe	199	37.8
Helplessness, despair		
Not at all	108	20.6
Mild	77	14.7
Moderate	130	24.8
Severe	95	18.1
Very severe	114	21.8
Exhaustion		
Not at all	55	10.5
Mild	62	11.8
Moderate	149	28.4
Severe	110	21.0
Very severe	148	28.2
Worry		
Not at all	10	1.9
Mild	18	3.4
Moderate	53	10.2
Severe	130	24.9
Very severe	311	59.6
Compassion		
Not at all	8	1.5
Mild	4	0.8
Moderate	30	5.7
Severe	82	15.6
Very severe	402	76.4
Fear to get infected		
Not at all	265	50.4
Mild	91	17.3
Moderate	75	14.3
Severe	44	8.4
Very severe	51	9.7
Guilt		
Not at all	199	38.0
Mild	82	15.6
Moderate	94	17.9
Severe	60	11.5
Very severe	89	17.0
Social burden		
Changes in daily routine		
Yes	413	79.0
No	110	21.0
Economic burden		
Days off work		
None	173	33.1
1–2	117	22.4
3–4	96	18.4
5+	76	14.5
Not employed	61	11.7
Other expenditures		
Yes	380	75.2
No	125	24.8

^a^The sum of the stratified numbers can differ according to the parameters due to missing values

### Factors associated with the impact of the disease on the family quality of life

To evaluate the emotional burden of the disease, the three most common indicators of emotional burden were chosen for the further analysis, i.e., compassion, worry and stress/anxiety. To better perceive and interpret the data for further analysis the categories “severe” and “very severe” were combined, and the categories “mild” and “not at all” were combined.

In Table 4 (uploaded as separate file) the independent factors (sociodemographic, subjective and objective health status indicators) associated with the emotional burden of the disease are summarized.

**Table 4:** Emotional burden (stress/anxiety, worry, compassion) of the disease stratified by the associated factors (*n* = 527^a^)

Factor	Not at all / mild		Moderate		Severe / very severe		*p*
	Number	%	Number	%	Number	%	
STRESS / ANXIETY							
*Sociodemographic factors*							
Gender							
Female	33	12.8	46	17.9	178	69.3	0.16
Male	28	10.4	66	24.5	175	65.1	
Age							
≤ 12 months	15	9.6	37	23.7	104	66.7	0.24
13–24 months	16	9.5	34	20.2	118	70.2	
25–36 months	9	10.2	21	23.9	58	65.9	
37+ months	21	18.6	20	17.7	72	63.7	
Age of the mother							
≤ 24 years	5	9.1	12	21.8	38	69.1	0.78
25–34 years	35	10.4	73	21.8	227	67.8	
35–44 years	20	15.9	24	19.0	82	65.1	
45+ years	1	12.5	2	25.0	5	62.5	
Age of the father							
≤ 24 years	2	7.4	7	25.9	18	66.7	0.99
25–34 years	35	12.5	59	21.0	187	65.5	
35–44 years	19	11.6	35	21.3	110	67.1	
45+ years	4	10.8	8	21.6	25	67.6	
Education of the mother							
Primary	4	13.8	5	17.2	20	69.0	0.95
Secondary / vocational	23	12.2	38	20.1	128	67.7	
Higher	34	11.2	67	22.1	202	66.7	
Education of the father							
Primary	7	19.4	6	16.7	23	63.9	0.57
Secondary / vocational	25	10.2	55	22.5	164	67.2	
Higher	28	12.6	47	21.2	147	66.2	
Family structure							
Both parents	58	11.8	106	21.6	327	66.6	0.20
Single parent	1	3.6	4	14.3	23	82.1	
Place of residence							
Urban	55	12.3	96	21.4	297	66.3	0.74
Rural	6	9.1	14	21.2	46	69.7	
*Objective evaluation of the health status*							
Vomiting (times per 24 h)							
0	1	33.3	0	0	2	66.7	0.36
1	12	12.4	18	18.6	67	69.1	
2	28	12.4	58	25.7	140	61.9	
3	18	10.8	30	18.0	119	71.3	
Diarrhea (times per 24 h)							
1	9	14.3	12	19.0	42	66.7	0.95
2	13	10.6	27	22.0	83	67.5	
3	37	11.9	68	21.8	207	66.3	
Severity of episode (Vesikari)							
Moderate	4	11.4	6	17.1	25	71.4	0.79
Severe / very severe	55	11.9	101	21.9	306	66.2	
*Subjective evaluation of the health status*							
Severity of diarrhea							
Not at all / mild	5	15.2	5	15.2	23	69.7	0.41
Moderate	13	17.1	17	22.4	46	60.5	
Severe / very severe	43	10.4	89	21.6	280	68.0	
Severity of vomiting							
Not at all / mild	15	10.5	35	24.5	93	65.0	0.33
Moderate	6	6.9	21	24.1	60	69.0	
Severe / very severe	40	13.7	56	19.2	195	67.0	
Severity of fever							
Not at all / mild	22	15.4	44	30.8	77	53.8	0.003
Moderate	10	9.2	22	20.2	77	70.6	
Severe / very severe	29	10.9	45	16.9	193	72.3	
Severity of abdominal pain							
Not at all / mild	24	14.7	34	20.9	105	64.4	0.61
Moderate	14	10.4	32	23.9	88	65.7	
Severe / very severe	21	10.0	44	21.0	145	69.0	
Severity of insufficient fluid intake							
Not at all / mild	8	10.8	16	21.6	50	67.6	0.74
Moderate	11	13.1	22	26.2	51	60.7	
Severe / very severe	42	11.6	73	20.1	248	68.3	
Severity of loss of appetite							
Not at all / mild	13	14.6	19	21.3	57	64.0	0.07
Moderate	9	8.5	33	31.1	64	60.4	
Severe / very severe	39	12.1	59	18.3	224	69.6	
Severity of apathy							
Not at all / mild	7	8.2	20	23.5	58	68.2	0.37
Moderate	17	16.0	25	23.6	64	60.4	
Severe / very severe	36	11.2	64	19.9	222	68.9	
Severity of inflamed bottom							
Not at all / mild	38	13.4	66	23.2	180	63.4	0.35
Moderate	10	11.6	14	16.3	62	72.1	
Severe / very severe	13	8.8	30	20.4	104	70.7	
Severity of interrupted sleep mode							
Not at all / mild	36	13.8	54	20.8	170	65.4	0.19
Moderate	14	11.6	32	26.4	75	62.0	
Severe / very severe	11	8.0	25	18.2	101	73.7	
Severity of tearfulness							
Not at all / mild	35	22.9	30	19.6	88	57.5	< 0.001
Moderate	12	7.9	43	28.3	97	63.8	
Severe / very severe	14	6.5	39	18.2	161	75.2	
Severity of anxiety / irritability							
Not at all / mild	44	20.2	47	21.6	127	58.3	< 0.001
Moderate	9	7.4	36	29.8	76	62.8	
Severe / very severe	8	4.5	27	15.2	143	80.3	
WORRY							
*Sociodemographic factors*							
Gender							
Female	12	4.7	20	7.8	224	87.5	0.16
Male	16	6.0	33	12.4	217	81.6	
Age							
≤ 12 months	6	3.9	16	10.4	132	85.7	0.12
13–24 months	6	3.6	15	8.9	147	87.5	
25–36 months	5	5.7	6	6.9	76	87.4	
37+ months	11	9.8	16	14.3	85	75.9	
Age of mother							
≤ 24 years	4	7.3	1	1.8	50	90.9	0.21
25–34 years	15	4.5	35	10.5	283	85.0	
35–44 years	9	7.3	14	11.3	101	81.5	
45+ years	0	0	2	25.0	6	75.0	
Age of father							
≤ 24 years	1	3.7	1	3.7	25	92.6	0.81
25–34 years	15	5.4	27	9.6	238	85.0	
35–44 years	9	5.6	18	11.1	135	83.3	
45+ years	1	2.8	5	13.9	30	83.3	
Education of mother							
Primary	0	0	2	6.9	27	93.1	0.58
Secondary / vocational	11	5.9	16	8.6	159	85.5	
Higher	17	5.6	33	10.9	252	83.4	
Education of father							
Primary	3	8.3	0	0	33	91.7	0.30
Secondary / vocational	12	5.0	26	10.8	203	84.2	
Higher	11	5.0	24	10.9	186	84.2	
Family structure							
Both parents	26	5.3	49	10.0	413	84.6	0.92
Single parent	1	3.6	3	10.7	24	85.7	
Place of residence							
Urban	25	5.6	48	10.8	372	83.6	0.27
Rural	3	4.6	3	4.6	59	90.8	
*Objective evaluation of the health status*							
Vomiting (times per 24 h)							
0	1	33.3	0	0	2	66.7	0.21
1	6	6.3	6	6.3	84	87.5	
2	11	4.9	26	11.6	187	83.5	
3	6	3.6	19	11.4	141	84.9	
Diarrhea (times per 24 h)							
1	6	9.5	3	4.8	54	85.7	0.13
2	5	4.1	10	8.1	108	87.8	
3	13	4.2	38	12.3	257	83.4	
Severity of episode (Vesikari)							
Moderate	2	5.7	2	5.7	31	88.6	0.64
Severe / very severe	22	4.8	49	10.7	387	84.5	
*Subjective evaluation of the health status*							
Severity of diarrhea							
Not at all / mild	2	6.1	5	15.2	26	78.8	0.07
Moderate	9	11.8	6	7.9	61	80.3	
Severe / very severe	17	4.2	41	10.0	350	85.8	
Severity of vomiting							
Not at all / mild	7	4.9	13	9.2	122	85.9	0.09
Moderate	1	1.1	14	16.1	72	82.8	
Severe / very severe	20	6.9	25	8.7	243	84.4	
Severity of fever							
Not at all / mild	11	7.8	13	9.2	117	83.0	0.14
Moderate	5	4.6	17	15.7	86	79.6	
Severe / very severe	12	4.5	22	8.3	232	87.2	
Severity of abdominal pain							
Not at all / mild	13	8.0	18	11.0	132	81.0	0.34
Moderate	8	6.1	14	10.7	109	83.2	
Severe / very severe	7	3.3	19	9.1	183	87.6	
Severity of insufficient fluid intake							
Not at all / mild	5	6.8	6	8.1	63	85.1	0.89
Moderate	3	3.6	9	10.7	72	85.7	
Severe / very severe	20	5.6	37	10.3	302	84.1	
Severity of loss of appetite							
Not at all / mild	5	5.6	9	10.1	75	84.3	0.57
Moderate	3	2.9	14	13.5	87	83.7	
Severe / very severe	19	5.9	29	9.0	273	85.0	
Severity of apathy							
Not at all / mild	1	1.2	12	14.3	71	84.5	0.24
Moderate	7	6.6	12	11.3	87	82.1	
Severe / very severe	19	5.9	28	8.8	273	85.3	
Severity of inflamed bottom							
Not at all / mild	20	7.1	34	12.0	229	80.9	0.13
Moderate	4	4.7	5	5.8	77	89.5	
Severe / very severe	4	2.8	13	9.0	128	88.3	
Severity of interrupted sleep mode							
Not at all / mild	19	7.4	26	10.1	213	82.6	0.33
Moderate	5	4.2	14	11.7	101	84.2	
Severe / very severe	4	2.9	12	8.8	121	88.3	
Severity of tearfulness							
Not at all / mild	16	10.5	19	12.5	117	77.0	0.006
Moderate	6	4.0	16	10.6	129	85.4	
Severe / very severe	6	2.8	16	7.5	191	89.7	
Severity of anxiety / irritability							
Not at all / mild	21	9.7	26	12.0	170	78.3	< 0.001
Moderate	2	1.7	19	15.8	99	82.5	
Severe / very severe	5	2.8	7	4.0	165	93.2	
COMPASSION							
*Sociodemographic factors*							
Gender							
Female	3	1.2	15	5.8	239	93.0	0.25
Male	9	3.3	15	5.6	245	91.1	
Age							
≤ 12 months	3	1.9	4	2.6	149	95.5	0.21
13–24 months	6	3.6	9	5.4	153	91.1	
25–36 months	1	1.1	6	6.8	81	92.0	
37+ months	2	1.8	11	9.7	100	88.5	
Age of mother							
≤ 24 years	1	1.8	1	1.8	53	96.4	0.68
25–34 years	8	2.4	17	5.1	310	92.5	
35–44 years	3	2.4	10	7.9	113	89.7	
45+ years	0	0	1	12.5	7	87.5	
Age of father							
≤ 24 years	0	0	1	3.7	26	96.3	0.43
25–34 years	7	2.5	11	3.9	263	93.6	
35–44 years	5	3.0	11	6.7	148	90.2	
45+ years	0	0	4	10.8	33	89.2	
Education of mother							
Primary	0	0	1	3.4	28	96.6	0.36
Secondary / vocational	7	3.7	8	4.2	174	92.1	
Higher	5	1.7	20	6.6	278	91.7	
Education of father							
Primary	4	11.1	2	5.6	30	83.3	0.01
Secondary / vocational	4	1.6	13	5.3	227	93.0	
Higher	4	1.8	11	5.0	207	93.2	
Family structure							
Both parents	12	2.4	27	5.5	452	92.1	0.67
Single parent	0	0	2	7.1	26	92.9	
Place of residence							
Urban	11	2.5	27	6.0	410	91.5	0.79
Rural	1	1.5	3	4.5	62	93.9	
*Objective evaluation of the health status*							
Vomiting (times per 24 h)							
0	0	0	0	0	3	100.0	0.22
1	4	4.1	10	10.3	83	85.6	
2	4	1.8	9	4.0	213	94.2	
3	2	1.2	10	6.0	155	92.8	
Diarrhea (times per 24 h)							
1	1	1.6	6	9.5	56	88.9	0.54
2	4	3.3	6	4.9	113	91.9	
3	5	1.6	17	5.4	290	92.9	
Severity of episode (Vesikari)							
Moderate	0	0	4	11.4	31	88.6	0.24
Severe / very severe	10	2.2	25	5.4	427	92.4	
*Subjective evaluation of the health status*							
Severity of diarrhea							
Not at all / mild	0	0	2	6.1	31	93.9	0.48
Moderate	3	3.9	2	2.6	71	93.4	
Severe / very severe	8	1.9	26	6.3	378	91.7	
Severity of vomiting							
Not at all / mild	2	1.4	12	8.4	129	90.2	0.32
Moderate	3	3.4	2	2.3	82	94.3	
Severe / very severe	6	2.1	16	5.5	269	92.4	
Severity of fever							
Not at all / mild	3	2.1	11	7.7	129	90.2	0.84
Moderate	2	1.8	6	5.5	101	92.7	
Severe / very severe	6	2.2	13	4.9	248	92.9	
Severity of abdominal pain							
Not at all / mild	6	3.7	12	7.4	145	89.0	0.31
Moderate	1	0.7	6	4.5	127	94.8	
Severe / very severe	4	1.9	10	4.8	196	93.3	
Severity of insufficient fluid intake							
Not at all / mild	0	0	5	6.8	69	93.2	0.41
Moderate	2	2.4	2	2.4	80	95.2	
Severe / very severe	9	2.5	23	6.3	331	91.2	
Severity of loss of appetite							
Not at all / mild	2	2.2	4	4.5	83	93.3	0.98
Moderate	2	1.9	6	5.7	98	92.5	
Severe / very severe	7	2.2	20	6.2	295	91.6	
Severity of apathy							
Not at all / mild	3	3.5	7	8.2	75	88.2	0.60
Moderate	3	2.8	5	4.7	98	92.5	
Severe / very severe	5	1.6	18	5.6	299	92.9	
Severity of inflamed bottom							
Not at all / mild	6	2.1	22	7.7	256	90.1	0.12
Moderate	3	3.5	4	4.7	79	91.9	
Severe / very severe	2	1.4	3	2.0	142	96.6	
Severity of interrupted sleep mode							
Not at all / mild	4	1.5	16	6.2	240	92.3	0.73
Moderate	4	3.3	5	4.1	112	92.6	
Severe / very severe	3	2.2	9	6.6	125	91.2	
Severity of tearfulness							
Not at all / mild	3	2.0	12	7.8	138	90.2	0.46
Moderate	5	3.3	7	4.6	140	92.1	
Severe / very severe	3	1.4	10	4.7	201	93.9	
Severity of anxiety / irritability							
Not at all / mild	4	1.8	15	6.9	199	91.3	0.53
Moderate	3	2.5	9	7.4	109	90.1	
Severe / very severe	4	2.2	6	3.4	168	94.4	

None of the sociodemographic factors showed a significant association with the indicators of emotional burden of rotavirus infection. The only factor showing a significant association with compassion was education of the father, i.e., fathers with higher education corresponded to a higher proportion reporting high or very high levels of compassion (*p* = 0.01).

None of the indicators of emotional burden showed a statistically significant association with the objective health status variables as well as with most of the subjective indicators of the child’s health status. A significant correlation was found only between stress/anxiety and fever (more severe fever corresponded to a higher level of severe stress/anxiety (*p* = 0.003)), between stress/anxiety and irritability of the child, between worry and irritability of the child (more intense irritability corresponded to a higher proportion of caregivers reporting severe or very severe stress (*p* < 0.001) or feelings of worry (*p* < 0.001)), and between stress or worry and tearfulness of the child (more severe tearfulness corresponded to a higher proportion of parents reporting severe or very severe stress (*p* < 0.001) or worry (*p* = 0.006)).

Table 5 (find uploaded as separate file) shows the social burden of the acute rotavirus infection and its associations with different independent variables. No statistically significant associations were found between the necessity to introduce changes in the caregiver’s daily routine and the objective health status indicators. The social burden showed statistically significant associations with different sociodemographic factors - older age of the child (*p* < 0.001), older age of the mother (*p* < 0.001) or the father (*p* = 0.03) and higher education level of the mother (*p* < 0.001) corresponded to larger proportions of caregivers reporting a need to introduce changes in their daily routine because of the rotavirus infection (such as sporting, educational or culture events/activities).

**Table 5:** Social burden (changes in daily routine) of the disease stratified by the associated factors (*n* = 527)

Factor	Yes		No		*p*
	Number	%	Number	%	
*Sociodemographic factors*					
Gender					
Female	211	82.4	45	17.6	0.06
Male	202	75.7	65	24.3	
Age					
≤ 12 months	105	67.7	50	32.3	< 0.001
13–24 months	126	75.4	41	24.6	
25–36 months	79	89.8	9	10.2	
37+ months	102	91.1	10	8.9	
Age of the mother (years)					
≤ 24 years	31	56.4	24	43.6	< 0.001
25–34 years	264	79.3	69	20.7	
35–44 years	111	88.8	14	11.2	
45+ years	5	62.5	3	37.5	
Age of the father (years)					
≤ 24 years	18	66.7	9	33.3	0.03
25–34 years	212	75.4	69	24.6	
35–44 years	137	85.1	24	14.9	
45+ years	32	86.5	5	13.5	
Education of the mother					
Primary	18	62.1	11	37.9	< 0.001
Secondary / vocational	133	71.1	54	28.9	
Higher	257	85.1	45	14.9	
Education of the father					
Primary	24	70.6	10	29.4	0.30
Secondary / vocational	190	78.2	53	21.8	
Higher	181	81.5	41	18.5	
Family structure					
Both parents	386	79.1	102	20.9	0.61
Single parent	21	75.0	7	25.0	
Place of residence					
Urban	350	78.5	96	21.5	0.57
Rural	53	81.5	12	18.5	
*Objective evaluation of the health status*					
Vomiting (times per 24 h)					
0	1	33.3	2	66.7	0.08
1	70	72.2	27	27.8	
2	178	79.1	47	20.9	
3	135	81.3	31	18.7	
Diarrhea (times per 24 h)					
1	48	77.4	14	22.6	0.07
2	87	71.3	35	28.7	
3	254	81.4	58	18.6	
Severity of episodes (Vesikari)					
Moderate	24	68.6	11	31.4	0.13
Severe / very severe	366	79.6	94	20.4	
*Subjective evaluation of the health status*					
Severity of diarrhea					
Not at all / mild	24	72.7	9	27.3	0.36
Moderate	56	74.7	19	25.3	
Severe / very severe	330	80.3	81	19.7	
Severity of vomiting					
Not at all / mild	110	77.5	32	22.5	0.23
Moderate	63	73.3	23	26.7	
Severe / very severe	237	81.4	54	18.6	
Severity of fever					
Not at all / mild	102	71.8	40	28.2	0.02
Moderate	94	85.5	16	14.5	
Severe / very severe	215	80.8	51	19.2	
Severity of abdominal pain					
Not at all / mild	123	75.9	39	24.1	0.63
Moderate	105	78.9	28	21.1	
Severe / very severe	168	80.0	42	20.0	
Severity of insufficient fluid intake					
Not at all / mild	50	68.5	23	31.5	0.04
Moderate	63	76.8	19	23.2	
Severe / very severe	296	81.5	67	18.5	
Severity of loss of appetite					
Not at all / mild	64	72.2	24	27.3	0.06
Moderate	78	74.3	27	25.7	
Severe / very severe	264	82.2	57	17.8	
Severity of apathy					
Not at all / mild	60	71.4	24	28.6	0.14
Moderate	85	81.0	20	19.0	
Severe / very severe	260	81.0	61	19.0	
Severity of inflamed bottom					
Not at all / mild	219	77.9	62	22.1	0.60
Moderate	82	82.8	15	17.2	
Severe / very severe	117	80.1	29	19.9	
Severity of interrupted sleep mode					
Not at all / mild	199	77.1	59	22.9	0.48
Moderate	96	79.3	25	20.7	
Severe / very severe	112	82.4	24	17.6	
Severity of tearfulness					
Not at all / mild	116	76.3	36	23.7	0.59
Moderate	123	80.9	29	19.1	
Severe / very severe	169	79.7	43	20.3	
Severity of anxiety / irritability					
Not at all / mild	168	77.4	49	22.6	0.64
Moderate	98	81.0	23	19.0	
Severe / very severe	142	80.7	34	19.3	

Out of all subjective health status indicators, only fever (similarly to the emotional burden) and insufficient fluid intake were significantly associated with the social burden of the disease. That is, a larger proportion of caregivers reported needing to introduce changes in their daily routine when their child had more severe fevers (*p* = 0.02) or insufficient fluid intake (*p* = 0.04).

Finally, Table 6 (find uploaded as separate file) reveals the factors that increased the economic burden of rotavirus infection. None of the objective health status indicators significantly influenced the working abilities of the parents. Only two sociodemographic factors showed a significant impact on the economic burden of the disease: a higher age of the child (*p* = 0.01) and higher level of education of the mother (*p* = 0.02) corresponded to a larger proportion of respondents reporting the need to be absent from work for at least 1 day.

**Table 6:** Economic burden (days off work) of the disease stratified by the associated factors (*n* = 527^a^)

Factor	None		At least one		Not employed		*p*
	Number	%	Number	%	Number	%	
*Sociodemographic factors*							
Gender							
Female	81	31.6	148	57.8	27	10.5	0.49
Male	92	34.5	141	52.8	34	12.7	
Age							
≤ 12 months	67	43.8	61	39.9	25	16.3	0.01
13–24 months	56	33.3	96	57.1	16	9.5	
25–36 months	22	24.7	57	64.0	10	11.2	
37+ months	28	25.0	74	66.1	10	8.9	
Age of the mother							
≤ 24 years	17	31.5	28	51.9	9	16.7	0.84
25–34 years	110	32.9	188	56.3	36	10.8	
35–44 years	41	32.8	69	55.2	15	12.0	
45+ years	4	50.0	3	37.5	1	12.5	
Age of the father							
≤ 24 years	11	42.3	11	42.3	4	15.4	0.48
25–34 years	95	33.9	152	54.3	33	11.8	
35–44 years	54	33.1	92	56.4	17	10.4	
45+ years	7	18.9	25	67.6	5	13.5	
Education of the mother							
Primary	14	48.3	12	41.4	3	10.3	0.02
Secondary / vocational	73	39.0	85	45.5	29	15.5	
Higher	85	28.1	188	62.3	29	9.6	
Education of the father							
Primary	12	35.3	16	47.1	6	17.6	0.41
Secondary / vocational	82	33.6	140	57.4	22	9.0	
Higher	71	32.1	120	54.3	30	13.6	
Family structure							
Both parents	159	32.6	271	55.5	58	11.9	0.64
Single parent	11	39.3	15	53.6	2	7.1	
Place of residence							
Urban	145	32.6	245	55.1	55	12.4	0.53
Rural	23	34.8	38	57.6	5	7.6	
*Objective evaluation of the health status*							
Vomiting (times per 24 h)							
0	3	1000.	0	0	0	0	0.28
1	32	33.3	50	52.1	14	14.6	
2	78	34.7	123	54.7	24	10.7	
3	52	31.1	95	56.9	20	12.0	
Diarrhea (times per 24 h)							
1	23	36.5	33	52.4	7	11.1	0.95
2	40	32.5	66	53.7	17	13.8	
3	104	33.5	170	54.8	36	11.6	
Severity of episodes (Vesikari)							
Moderate	14	40.0	17	48.6	4	11.4	0.70
Severe / very severe	152	33.0	252	54.8	56	12.2	
*Subjective evaluation of the health status*							
Severity of diarrhea							
Not at all / mild	13	39.4	17	51.5	3	9.1	0.43
Moderate	31	40.8	39	51.3	6	7.9	
Severe / very severe	128	31.2	232	56.6	50	12.2	
Severity of vomiting							
Not at all / mild	55	38.7	71	50.0	16	11.3	0.26
Moderate	27	31.0	46	52.9	14	16.1	
Severe / very severe	90	30.9	171	58.8	30	10.3	
Severity of fever							
Not at all / mild	54	38.0	70	49.3	18	12.7	0.19
Moderate	27	24.8	69	63.3	13	11.9	
Severe / very severe	90	33.7	150	56.2	27	10.1	
Severity of abdominal pain							
Not at all / mild	61	37.4	84	51.5	18	11.0	0.12
Moderate	51	38.3	71	53.4	11	8.3	
Severe / very severe	57	27.1	124	59.0	29	13.8	
Severity of insufficient fluid intake							
Not at all / mild	32	43.8	28	38.4	13	17.8	0.02
Moderate	30	35.7	43	51.2	11	13.1	
Severe / very severe	110	30.4	216	59.7	36	9.9	
Severity of loss of appetite							
Not at all / mild	39	44.3	39	44.3	10	11.4	0.06
Moderate	39	36.8	54	50.9	13	12.3	
Severe / very severe	93	29.0	192	59.8	36	11.2	
Severity of apathy							
Not at all / mild	33	39.8	39	47.0	11	13.3	0.20
Moderate	34	32.4	55	52.4	16	15.2	
Severe / very severe	99	30.7	192	59.4	32	9.9	
Severity of inflamed bottom							
Not at all / mild	81	28.7	174	61.7	27	9.6	0.03
Moderate	37	42.5	38	43.7	12	13.8	
Severe / very severe	53	36.3	74	50.7	19	13.0	
Severity of interrupted sleep mode							
Not at all / mild	89	34.4	148	57.1	22	8.5	0.33
Moderate	37	30.6	66	54.5	18	14.9	
Severe / very severe	45	33.1	72	52.9	19	14.0	
Severity of tearfulness							
Not at all / mild	53	34.9	80	52.6	19	12.5	0.92
Moderate	47	30.7	88	57.5	18	11.8	
Severe / very severe	71	33.5	118	55.7	23	10.8	
Severity of anxiety / irritability							
Not at all / mild	69	31.7	121	55.5	28	12.8	0.27
Moderate	34	28.1	76	62.8	11	9.1	
Severe / very severe	67	38.1	90	51.1	19	10.8	

^a^The sum of the stratified numbers can differ according to the parameters due to missing values

Out of all subjective health status indicators, only insufficient fluid intake (like the social burden) and inflamed bottom seems to increase the economic burden of the infection. A larger proportion of caregivers reported the need to be absent from work for cases of more severe insufficient fluid intake (*p* = 0.02) or inflamed bottom (*p* = 0.03) of their child.

Therefore, it can be concluded that the objective health status of the child does not influence the emotional, social or economic burden of the rotavirus infection, whereas the parents’ subjective perceptions of the child’s health status and some sociodemographic characteristics, such as the age of the child and the age or education of parents do influence the burden.

## Discussion

This study reveals the impact of rotavirus gastroenteritis on HRQL of families whose children are affected. As the disease is characterized by a sudden onset, it can disrupt daily routine, require unexpected changes, and thus, can affect the physical, emotional and social wellbeing of the child and family. The results show that an acute illness negatively effects the family and increases their emotional, social and economic disease burden. Parents reported moderate or severe parental distress, worry and anxiety, as well as intense feelings of an exhaustion, helplessness and despair. This is consistent with the results of other studies that also reported parental emotions and feelings due to a child’s illness. Parents reported high distress levels during the episode of rotavirus gastroenteritis [5, 17, 18] and felt exhausted and helpless [18]. Our study concludes that parents of hospitalized children are faced with disruptions of their daily routine and social activities. This fact has also been established in similar studies [17]. The economic burden of disease is related to lost days of work and additional expenditures. In our study and other studies, parents experienced lost work days [5, 20] and additional expenditures. [17, 21].

Current research has shown that stress, anxiety, worry and compassion are the most often (and more intense) feelings experienced by parents due a child’s illness. Based on a subjective assessment of disease symptoms, parents reported that severe fever of the child, irritability and tearfulness promoted higher parental stress levels. Emotional reactions, to a certain extent, are socially formatted and structured [22]. Parental responses to a child’s symptoms and their subsequent emotional feelings can be incorporated and interpreted in a cultural framework. In Latvia, fever in children is possibly overestimated as an abnormal and potentially life-threatening condition. This, in turn, can lead to excessive parental stress reactions. Cultural and personal beliefs held by parents also influence perceptions of how a “healthy child” should look and behave [23]. Tearfulness and irritability are usually not associated with the image of a healthy child in Latvia, and these symptoms can provoke more intense levels of parental distress, worry and anxiety. Cultural factors regarding the impact of rotavirus gastroenteritis on families were analyzed in an ethnographic study in Taiwan and Vietnam [21]; another study also compared the emotional reactions of Spanish, Italian and Polish parents due to childhood acute rotavirus gastroenteritis. To help parents manage their child’s health needs during an acute illness and their own perceptions and reactions toward their child’s symptoms, sufficient parental health education is required [24]. A successful and mutual physician-parent communication, as the foundation of the therapeutic relationship, is an essential tool for better social support [25]; otherwise, lack of communication with a child’s parents can lead to misunderstandings and cause additional stress. The social burden of disease is an essential domain of HRQL. This study revealed that older mothers and fathers more often reported the need to unexpectedly change their daily routine because of their child’s acute illness, which was also true for mothers with higher education levels. This finding could be explained by the group of parents aged 35 or more as having more social duties and activities. Parents reported that severe fever and insufficient fluid intake were the most prevalent symptoms of their child that caused disruption of their daily schedule. This could be linked to cultural issues, parental education and health communication. In Latvia, information on child dehydration is broadly released, and the notion that children should drink fluids is strongly embodied in public discourses and practices.

Our study revealed that the main aspect of economic burden is the loss of work days. The larger proportion of parents (caregivers) experienced absence from work for at least 1 day due to a childhood rotavirus gastroenteritis when the child was of higher age. This finding could be explained by paid parental leave in Latvia, that covers first year of life. As children grow older*,* both parents usually are employed and sick*-*leave usually is required. Mothers with the higher educational levels more often reported the need to be absent from work at least 1 day. A possible explanation could be related to job specificity (duties, responsibility, etc.) and/or better social insurance and social security system. Parents reported that an inflamed bottom and insufficient fluid intake were the most prevalent symptoms of their child that led to lost work days, which could be linked to cultural and informational issues regarding symptom perception and management.

This study confirmed that acute childhood rotavirus gastroenteritis places a considerable burden on families. It affects all domains of HRQL. This study provides in-depth insight into parental subjective evaluation of their child’s symptoms and their reactions to these symptoms. These results are important for promoting better communication between physicians and parents.

Additional research may be necessary to identify more profound factors and to measure the associations among factors in considering the current development of conceptual frameworks for HRQL assessment in acute gastroenteritis [26].

This study has several limitations. First, the results are not fully generalizable, as only hospitalized children and their families were included. Thus, the results may not be relevant upon extrapolation to milder cases of rotavirus infection.

## Conclusions

In this study, we found that the objective health status of the child did not influence the emotional, social or economic burden of rotavirus infection, but rather parents’ subjective perceptions of their child’s health status and sociodemographic characteristics such as the age of the child or the age or education of parents did affect their burden.

A better understanding of how acute episode affect the child and the child’s family could help to ease parental fears and advice parents on the characteristics of rotavirus infection and the optimal care of an affected child.

## Abbreviations

CI: Confidence interval
HRQL: Health-related quality of life
n: Absolute number
OR: Odds ratio
PCR: Polymerase chain reaction
